# A Mixed Comparison of Interventions for Kinesiophobia in Individuals With Musculoskeletal Pain: Systematic Review and Network Meta-Analysis

**DOI:** 10.3389/fpsyg.2022.886015

**Published:** 2022-06-29

**Authors:** Jialu Huang, Yining Xu, Rongrong Xuan, Julien S. Baker, Yaodong Gu

**Affiliations:** ^1^Faculty of Sports Science, Ningbo University, Ningbo, China; ^2^The Affiliated Hospital of Medical School of Ningbo University, Ningbo, China; ^3^Department of Sport and Physical Education, Hong Kong Baptist University, Kowloon, Hong Kong SAR, China

**Keywords:** kinesiophobia, musculoskeletal pain, network meta-analysis, systematic review, non-surgical

## Abstract

**Objective:**

This systematic review aims to make a mixed comparison of interventions for kinesiophobia and individuals with musculoskeletal pain.

**Methods:**

A comprehensive search strategy was conducted in the database of PubMed, MEDLINE, and Web of Science with the inclusion criteria: (1) randomized controlled design; (2) patients with musculoskeletal pain as participants; (3) treatments protocols of kinesiophobia as interventions or comparisons; (4) the score of Tampa Scale Kinesiophobia (TSK) as outcome measures. A network meta-analysis was used to synthesize the data after checking the model consistency. The risk of bias was assessed by the Cochrane Collaboration Risk of Bias Assessment Tool.

**Results:**

Thirty-one studies were included in this review after a comprehensive search strategy with a low risk of bias and good consistency. According to the results of the network meta-analysis, a multi-modal treatment protocol had the highest probability to become the best choice in dealing with kinesiophobia caused by musculoskeletal pain, whereas psychological treatment protocols also showed a potentially positive effect on musculoskeletal pain-induced kinesiophobia.

**Conclusion:**

Multi-modal protocols could be recommended as the preferred option when dealing with kinesiophobia caused by musculoskeletal pain. However, it is still worth mentioning that there are also potentially positive therapeutic effects of psychological interventions. Since the concept of kinesiophobia is based on the fear-avoidance model, the psychological mechanism should be paid enough attention to during treatment.

**Registration Number:**

CRD42021286450.

## Introduction

Kinesiophobia, which was firstly proposed by Miller in 1990 based on an aspect of the fear-avoidance model, was a conceptual definition that could describe a fear of pain and an excessive, irrational, and debilitating fear to carry out a physical movement due to a feeling of vulnerability to a painful injury or re-injury (Kori et al., 2011; Vincent et al., 2013; Czuppon et al., 2014; Smith et al., 2014; Monticone et al., 2015; Urquhart et al., 2015; Zdziarski et al., 2015; Ferrándiz et al., 2016; Dvir et al., 2018; Weissenfels et al., 2019; Gholami et al., 2020; Östenberg et al., 2022; Xiang et al., 2022). The developing mechanism of kinesiophobia is multiple and complex, among which the failure and accidents in an individual's movements and the following physical and social activity are the most common. Individuals who are highly fear-avoidant believe that pain is a sign of bodily harm and that any activities causing pain are dangerous and should be avoided (Helmhout et al., 2010; Da Luz et al., 2014). Pain, which might be induced by musculoskeletal disorders, surgeries, and other traumatism, such as burns and scalds, is the most common feature in the progression of kinesiophobia (Galan-Martin et al., 2020; Riecke et al., 2020).

Kinesiophobia induced by musculoskeletal pain is most common and correlates with factors from biological, cognitive, and occupational perspectives (Luque-Suarez et al., 2019). Moreover, kinesiophobia might also occur and develop during the treatment and rehabilitation progression of musculoskeletal disorders, surgeries, and other traumatisms, which call for extra and special intervention (Archer et al., 2017).

The Tampa Scale for Kinesiophobia (TSK) and the Kinesiophobia Causes Scale (KCS) are two common tools being used in the assessment of kinesiophobia. The KCS, which is a questionnaire consisting of 20 close-ended questions with a range of scores from 0 to 100, is usually used to diagnose and determine the causes of motor passivity. Higher scores indicate a greater fear of movement. According to previous studies and evidence-based clinical practice, the TSK is mostly recommended in the assessment of kinesiophobia. For example, a systematic review published in 2004 mentioned that the overall score of TSK showed a significant positive correlation with the severity of the physical disability, and changes in TSK scores could be used as a comparative indicator of treatment effectiveness (Heuts et al., 2004). The original version of TSK is a 17-item questionnaire with the ratings available for each item: (1) disagree; (2) partially disagree; (3) partially agree; (4) strongly agree. The score of each item varies from 1 to 4 or 0 to 3. The score of each item would be summed after the participant finishes the assessment and the overall score ranges from 17 to 68 or from 0 to 51 (Roelofs et al., 2004; Damsgard et al., 2007; Lundberg et al., 2009; Mintken et al., 2010). There are also some shortened versions of TSK with high reliabilities and validities, such as TSK-13 and TSK-11, which have been widely used in the assessment of kinesiophobia caused by pain from other disorders and diseases, such as temporomandibular disorders (TSK-TMD) and chronic low back pain (TSK-CLBP) (Woby et al., 2005; Hapidou et al., 2012; Tkachuk and Harris, 2012; Larsson et al., 2014; Neblett et al., 2016). Moreover, TSK has also been translated into many different languages and has been identified with high test-retest reliability and internal consistency.

The choice of intervention treatment protocol options is an essential part of the treatment for musculoskeletal disorders. Studies of intervention treatment protocols for some musculoskeletal disorders have also found that certain treatments have a positive effect on kinesiophobia caused by pain from these musculoskeletal disorders (Vihstadt et al., 2014; Javdaneh et al., 2020; Tagliaferri et al., 2020). However, since most of the treatment protocols for musculoskeletal disorders are mainly focused on the injured tissues, aiming to restore the physiological functional integrity of damaged tissues, the TSK scores were usually reported as a secondary outcome, and there was a limited number of studies that focus on the treatments for kinesiophobia (Lara-Palomo et al., 2013; Monticone et al., 2017; Sarig Bahat et al., 2018). A systematic review and meta-analysis published in 2020, which only included randomized controlled trials that used the TSK-17 as outcome measures, identified the effect of multi-modal protocols that combined physical and psychological therapies for kinesiophobia caused by musculoskeletal disorders compared with uni-modal therapy of only phycological therapy or psychological therapy, demonstrating that, despite a large heterogeneity within studies, multi-modal protocols might be more effective in reducing kinesiophobia than the unimodal of only physical or psychological therapy (Xu et al., 2020). Therefore, a further review of studies in which more versions of TSK were used as outcome measures should be made to create more comprehensive, high-quality, and low-bias clinical evidence.

At present, there are numerous intervention treatment protocols for musculoskeletal disorders (Flores et al., 2017; Lenoir et al., 2019; Andersen et al., 2020). Therefore, it is not feasible to make an adjusted indirect comparison of interventions or the comparison of a mixed treatment that included every protocol. However, since some protocols just differ in operational details but are based on the same psychophysical principle and could be reclassified together, in this review, each treatment protocol in the studies included would be reclassified according to its operational characteristics and principles, and then put in the network meta-analysis.

According to previous studies, intervention treatment protocols for musculoskeletal disorders could be reclassified into passive modalities (PM), active physical exercise (APE), supervised training (ST), psychological intervention (PI), external-used devices (ED), treatment as usual (TAU), placebo treatment (Placebo), non-intervention (Blank), and multi-modal protocols (MP). In this review, the term PM is defined as therapist-led passive modalities such as acupuncture, manual massage, medication, and therapy using special equipment (laser, ultrasonic wave, magnet, thermal, etc.) (Heymans et al., 2006; Smeets et al., 2009; Ferrándiz et al., 2016; Saracoglu et al., 2020); the term APE refers to interventions in which participants perform physical training in an unsupervised environment or self-training according to a relevant program (resistance training or mobility training), relaxing, or proprioceptive training (Helmhout et al., 2004; Koumantakis et al., 2005; Gustavsson and von Koch, 2006; Pool et al., 2010; Nassif et al., 2011; Monticone et al., 2014; Miyamoto et al., 2016; Hotta et al., 2020); ST refers to physical training by the patient under the supervision of physiotherapists and caregivers (Hott et al., 2020; Meirelles et al., 2020). The term PI refers to treatments conducted by a professional psychologist or psychologist such as cognitive-behavioral therapy (CBT), meditation, and psychological interview (Lopez-Lopez et al., 2015; Monticone et al., 2016, 2018; Zdziarski-Horodyski et al., 2020; Sato et al., 2021), the term ED means the treatment protocol in which the participants would be asked to wear non-invasive therapy devices such as Kinesio tape, orthoses, or sportswear for a long-term (Castro-Sánchez et al., 2012), and the term MP refers to treatment protocols which include two or more protocols mentioned above, for example, physical exercise using VR devices or laser devices. It is important to note that two or more treatments of the same type are not part of a multi-modal protocols program, such as the use of Pilates combined with stretching body exercises in a sports modality (Meijer et al., 2006; Smith et al., 2019; Bahat et al., 2020; Gulsen et al., 2020; Tejera et al., 2020; Javdaneh et al., 2021).

Moreover, the category “Blank” in the reclassification of this review refers to keeping normal life or only receiving patient education with basic health advice (Nassif et al., 2011), whereas the category “TAU” refers to treatment as usual or usual care from nursing staff, and the category “Placebo” refers to sham therapy protocols such as sham manipulation and sham sustained natural apophyseal glides.

The objective of this systematic review is to make a mixed comparison of intervention treatment protocols on kinesiophobia for individuals with musculoskeletal pain by reclassifying treatment protocols from different perspectives according to the change in TSK scores and then to explore the most potential protocols.

## Methods

### Protocol and Registration

This systematic review was conducted according to the Preferred Reporting Items for Systematic Reviews and Meta-Analysis (PRISMA) guidelines (Moher et al., 2009). The eligibility and exclusion criteria and the search strategy were made and agreed upon by two authors (Jialu Huang and Yining Xu) with a priori to minimize bias. The PROSPERO Registration Number of this review is CRD42021286450.

### Eligibility Criteria (PICOS)

The eligibility criteria for inclusion were: (1) randomized controlled trials; (2) patients with musculoskeletal pain as participants; (3) intervention treatment protocols; (4) TAU, Placebo, or Blank protocols as comparisons; (5) the score of TSK as outcome measures.

### Exclusion Criteria

Studies were excluded if: (1) participants were patients with pain induced by non-musculoskeletal disorders, such as burns, traumatism, or surgeries; (2) intervention protocols contained surgical or invasive treatment such as surgeries and injections; (3) published abstract without full text or lack of data; (4) outcome measures did not correspond with those in the eligibility criteria; (5) not a randomized controlled trial, such as a cross-sectional study, case report, cohort study, and cross-over trial in a single group.

### Search Strategy

A comprehensive reproducible search strategy had been performed on the databases of PubMed, MEDLINE, and Web of Science from January 1990 to December 2021. Reference lists were also searched in all screened studies for identifying gray literature, which refers to the study that had not been openly published. If the data were insufficient, the authors were contacted and requested for missing data. The search terms used in each database were as follows: (1) in PubMed and Embase, the search term was “(kinesiophobia) AND [(randomized) OR (randomized)] [Titile/Abstract]”; (2) in Web of Science, the search term was “(AB kinesiophobia) AND (AB randomized OR randomized).”

### Risk of Bias Assessment

The risk of bias was assessed by two independent authors (Rongrong Xuan and Julien Baker) according to The Cochrane Collaboration Risk of Bias Assessment Tool (Armijo-Olivo et al., 2012). Every disagreement was discussed, and an independent arbitrator (Yaodong Gu) was invited when there was an agreement that could not be met.

### Data Extraction and Synthesis

All searched studies were imported into EndNote 20 (Thomson Reuters, Carlsbad, CA, USA) for further screening. Data were extracted by two independent authors (Jialu Huang and Yining Xu). Any discrepancies would be solved by an independent arbitrator (Yaodong Gu).

Detailed information of included studies was summarized and information such as participants' characteristics (age, gender, and type of musculoskeletal disorder) and details of intervention protocols with their reclassification were collected and put into an extraction sheet of summary of included studies. The data of each trial, which involved the version of TSK it used, sample size (N), mean value (Mean) with its standard deviation (SD) of each group in baseline, and every recording point would be extracted in an independent sheet for the data pre-processing.

### Data Pre-processing

Data pre-processing and analysis were conducted by two independent authors (Jialu Huang and Yining Xu). Microsoft Office Excel (Version 16.0, Microsoft Corporation, Redmond, WA, USA) was used to pre-process the original data by transferring all the outcomes into a uniform unit according to the clinical criteria. The Aggregate Data Drug Information System (ADDIS, Version 1.16.8, Produced by Drugis.org) was used to pool data into the network meta-analysis.

### Network Meta-Analysis

Since different versions of TSK have different total scores, the effect size of the network meta-analysis were presented in mean differences (MD) of the percentage of TSK score change. A network geometry was provided to display all kinds of interventions and key information such as the type of intervention represented by each node, direct comparisons between each pair of interventions represented by the edges, and the arms of each comparison which are represented by the number on every edge.

The random-effects standard deviations were calculated under both consistency and inconsistency models and compared with each other to identify if there was inconsistency within interventions. If there were closed loops in the intervention structure, the inconsistency of the evidence must be assessed. Moreover, while the results are easier to interpret, it requires a separate model to be run for each node to be split. The node-splitting analysis is an alternative method to assess inconsistency in network meta-analysis which assessed whether direct and indirect evidence on a specific node (the split node) agrees (Rouse et al., 2017).

The consistency model was used if there was neither closed-loop nor split node in the intervention structure, the random-effects standard deviations in the consistency and inconsistency models were identical, or the identified discrepancy could be determined by examining the calculating a respective Bayesian *p*-value in the node-splitting analysis was statistically insignificant (*p* > 0.05). Otherwise, the inconsistency model would be applied (Rouse et al., 2017).

When the consistency model was applied, the results were shown in the rank probability plot. The sum of all rank probabilities is 1, both within a rank over treatments and within a treatment over ranks. Besides, a league table was provided after the model of data analysis had been determined, reporting results that represented the mean difference in the column-defining treatment compared with the row-defining treatment. If the calculation was based on the inconsistency model, only a league table was provided after the model of data analysis had been determined (Catala-Lopez et al., 2014).

## Results

### Search Strategy and Information Extraction

**Figure 1:**
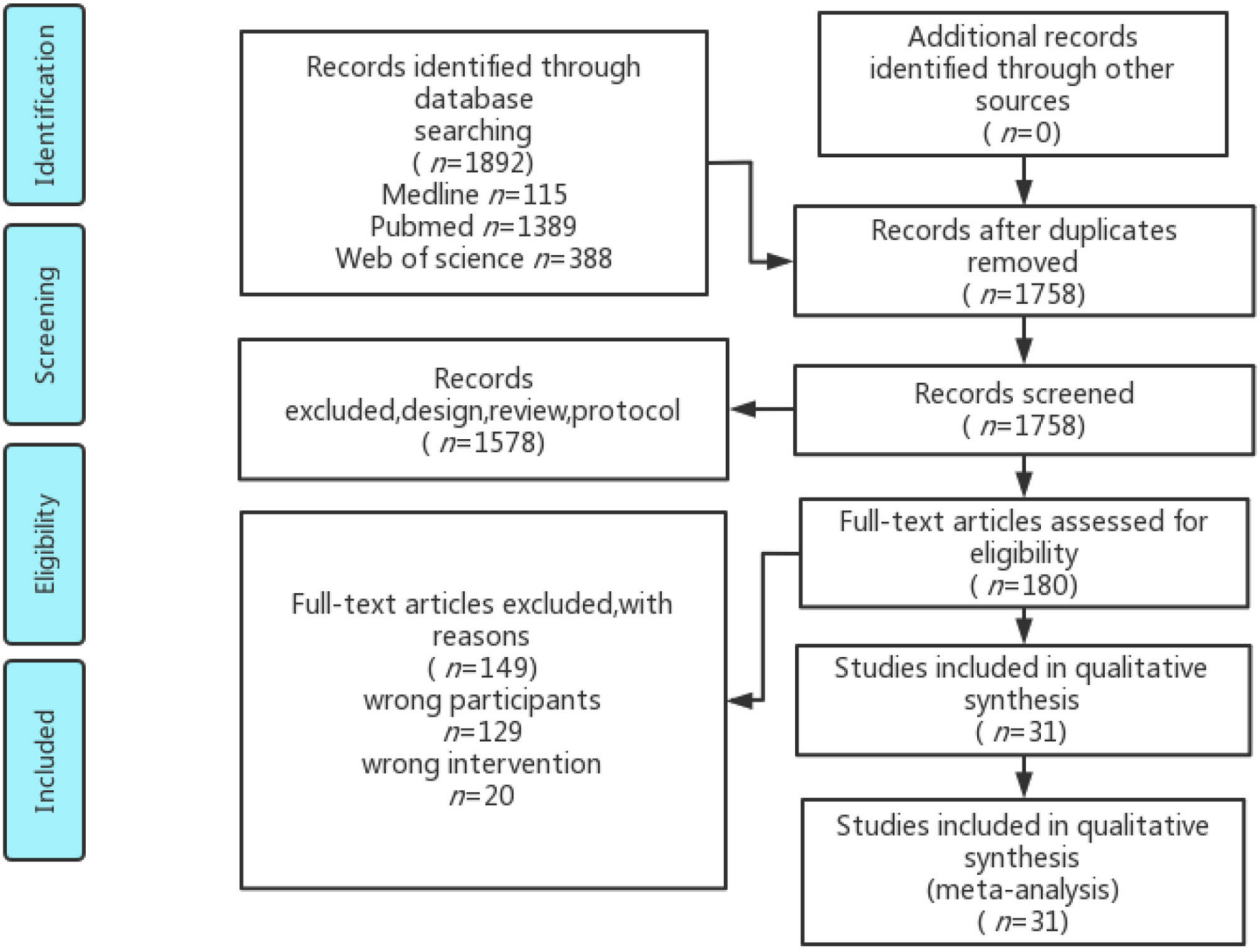
The PRISMA 2009 flow diagram of the search and study selection.

Thirty-one studies were included in the final analysis (Beltran-Alacreu et al., 2015; Hidalgo et al., 2015; Pires et al., 2015; Sarig Bahat et al., 2015; Castro-Sánchez et al., 2016; Pillastrini et al., 2016; Cruz-Díaz et al., 2017; Martínez-Cervera et al., 2017; Yilmaz Yelvar et al., 2017; Birinci et al., 2019; Gholami et al., 2020). The identification process is shown by a flow diagram in Figure 1 (Moher et al., 2009). The information of all included studies is presented in Table 1. All original data are provided in the Supplementary File.

**Table 1:** Detailed information of included studies.

**References**	**Participants**			**Interventions**			**Outcome (TSK Version)**
	**Type of pain**	**Age**	**Gender (female/all)**	**Treatment**	**Abstract of protocol**	**Category**	
Gustavsson and von Koch (2006)	Long-lasting neck pain	39.14	28/29	Applied Relaxation	The program contained 7 1.5-h sessions, over a period of 7 weeks. The sessions consisted of applied relaxation training, 4 body awareness exercises, and information about pain and stress management.	MP	TSK-17
				Treatment as usual	11 individual sessions spread over 20 weeks.	TAU	
Nassif et al. (2011)	Chronic lower back pain	45.23	32/75	Supervised active physical exercise program	60 min of physiotherapy and physical exercise 3 times a week for 2 months with full supervision by an in-house physiotherapist and physical educator.	MP	TSK-13
				No treatment	No direct intervention.	Blank	
Castro-Sánchez et al. (2012)	Chronic non-specific low back pain	48.50	40/59	Kinesio tape	Standardized Kinesio Tape application in sitting position. 4 blue-I placed at 25% tension overlapping in a star shape over the point of maximum pain in the lumbar area, 1 week.	ED	TSK-17
				Placebo Kinesio ape	A sham Kinesio Tape application, consisting of a single I-strip of the same tape applied transversely immediately above the point of maximum lumbar pain participants wore the tape, 1 week.	Placebo	
Lara-Palomo et al. (2013)	Chronic non-specific low back pain	48.50	41/61	Electro-massage	Electro-massage, over the lumbar and dorsal–lumbar region, 30 min at stimulation intensity of 30–50 mA, 2/week, 20 sessions.	PM	TSK-17
				Manual massage	Superficial manual massage session on the lower back area, 20 min, 2/week, 20 sessions.	PM	
Monticone et al. (2014)	Chronic low back pain	57.80	11/20	Spinal stabilizing exercises in addition to usual-care rehabilitation	Spinal stabilizing exercises in addition to usual-care rehabilitation (passive mobilization, stretching, and postural control), 60-min cognitive–behavioral sessions, 1/week, 8 weeks.	MP	TSK-13
				Usual-care rehabilitation	Routine care rehabilitation provided by the physiotherapist includes passive spinal movement, stretching, muscle strengthening and postural control. 60-min motor training sessions twice a week for 8 weeks.	ST	
Birinci et al. (2019)	Posttraumatic stiffness of the elbow	41.34	24/40	PNF stretching	hold-relax proprioceptive neuromuscular facilitation stretching combined with a structured exercise programme for posttraumatic stiffness of the elbow, 2/week, 6 weeks.	MP	TSK-17
				Static stretching	Static stretching combined with a structured exercise programme for posttraumatic stiffness of the elbow 0.2/week, 6 weeks.	MP	
Beltran-Alacreu et al. (2015)	Non-specific chronic neck pain	41.40	35/45	Manual therapy and therapeutic patient education	Therapeutic patient education in addition to manual treatment methods (specific passive movement of the cervical facet joints, overall movement of the cervical spine, and high-speed technology in the back area), 8 times in 4 weeks.	MP	TSK-11
				Manual therapy, therapeutic patient education, and a therapeutic exercise	Therapeutic patient education, therapeutic exercise, in addition to manual treatment methods (specific passive movement of the cervical facet joints, overall movement of the cervical spine, and high-speed technology in the back area), 8 times in 4 weeks.	MP	
				Manual therapy experimental	Specific passive movements in the facet cervical joints, global mobilization of the cervical spine, and high-velocity technique in the dorsal region. 8 times in 4 weeks.	PM	
Hidalgo et al. (2015)	Non-specific low back pain	39.25	16/32	Real lumbar Mulligan sustained natural apophyseal glides	Real lumbar Mulligan sustained natural apophyseal glides, 3 sets, 6 repetitions.	PM	TSK-17
				Sham lumbar Mulligan sustained natural apophyseal glides	Sham lumbar Mulligan sustained natural apophyseal glides, 3 sets, 6 repetitions.	Placebo	
Pires et al. (2015)	Chronic low back pain	51.00	40/62	Aquatic exercise and pain neurophysiology education	Water exercise programme under the supervision of a therapist, 6-week program consisting of 12 sessions + 2 sessions of pain neurophysiology education.	MP	TSK-13
				Aquatic exercise	Water exercise programme under the supervision of a therapist, 6-week program consisting of 12 sessions.	ST	
Sarig Bahat et al. (2015)	Chronic neck pain	40.90	22/32	Kinematic training	Home neck training method without physiotherapy supervision, 30 min each time, 4–6 times total, 5 weeks.	APE	TSK-17
				Kinematic and VR training	Neck training method with physiotherapist supervision and VR device, 30 min each time, 4–6 times total, 5 weeks.	MP	
Priore et al. (2020)	Patellofemoral pain	22.42	Unknown	Knee bracing	Participants received instructions to use the knee brace for 2 weeks while performing activities of daily living or sports that had previously resulted in knee pain.	ED	TSK-17
				Minimal intervention	Participants included in this group were instructed to do not use any type of orthoses, brace or bandage in the lower limbs for the period they were involved in the study. The knee brace was offered for the participants of this group after studies completion.	Blank	
Castro-Sanchez et al. (2016)	Chronic low back pain	45.00	39/62	Spinal manipulation	Side-lying pelvic girdle manipulation+Side-lying lumbar spine manipulation+Thoracic manipulation.	PM	TSK-17
				Functional technique	Patients in this group received a functional technique intervention targeted at the lumbosacral junction.	PM	
Castro-Sánchez et al. (2016)	Chronic non-specific low back pain	51.50	42/64	Craniosacral therapy	50-min Physical therapy, 1/week, 10 weeks.	PM	TSK-17
				Classic massage	30-min Classic massage, 1/week, 10 weeks.	PM	
Kurt et al. (2016)	Patellofemoral pain syndrome	31.27	49/84	Kinesio tape	2 days of Kinesio tape.	ED	TSK-17
				Placebo Kinesio tape	2 days of placebo Kinesio Tape.	Placebo	
Monticone et al. (2016)	Chronic low back pain	53.50	90/150	Cognitive-behavior therapy and exercise	1-h group-based cognitive-behavior therapy and physical training, 2/week, 5 weeks.	MP	TSK-13
				Traditional exercises	Patients engage in active physical activity, 1-h, 2/week, 5 weeks.	APE	
Pillastrini et al. (2016)	Chronic Non-specific Neck Pain	47.44	71/93	Global postural re-education and manual therapy	Manipulative massage and global postural re-education supervised by a physiotherapist, 1-h, 1–2/week, 9 times total.	MP	TSK-11
				Reference group	Manipulative massage under the supervision of a physiotherapist, 30 min, 9 times total.	PM	
Cruz-Díaz et al. (2017)	Chronic Low Back Pain on pain	36.25	63/98	Pilates training	Conscious patient-initiated Pilates mat training, 50-min, 2/week, 12 weeks.	APE	TSK-17
				Pilates training on mat	Conscious and active Pilates equipment training by the patient, 50-min, 2/week, 12 weeks.	APE	
				No treatment	No treatment.	Blank	
Martínez-Cervera et al. (2017)	Lateral epicondylalgia	51.94	13/24	Positive expectations	Manual therapy of mobilization with movements under positive expectations, 3/week, 48 h total.	PM	TSK-11
				Neutral expectations	Manual therapy of mobilization with movements under neutral expectations, 3/week, 48 h total.	PM	
Monticone et al. (2017)	Chronic neck pain	52.90	121/170	The multidisciplinary group	60-min physical training and 60-min psychologist interview/week, 10 weeks, and asked the patients to repeat the exercises at home.	MP	TSK-13
				General physiotherapy	The patient initiates 10 h of exercises including muscle strengthening, regional stretching and spinal mobilization.	APE	
Yilmaz Yelvar et al. (2017)	Non-specific low-back pain	49.55	28/44	Traditional physical therapy	Medication under the supervision of a physiotherapist.	PM	TSK-17
				Virtual walking integrated physiotherapy	15 min of intensive training with Hotpack and 15 min of ultrasound deep heating under the supervision of a physiotherapist. 5 sessions/week, 2 weeks in clinic, requested at home, 3 sessions/day.	MP	
Monticone et al. (2018)	Chronic neck pain	48.60	17/30	Normal cognitive-behavior therapy and physical exercise	1 week, 4 times of 60-min sessions of cognitive-behavioral therapy followed by 10 times 60-min sessions of exercises (2 sessions/week, 5 weeks).	MP	TSK-13
				Cognitive-behavior therapy based on TSK and physical exercise	1 week, 4 times of 60-min sessions of cognitive-behavioral therapy based on scores of Tampa scale of kinesiophobia followed by 10 times 60-min sessions of exercises, 2 sessions/week, 5 weeks total.	MP	
Pardo et al. (2018)	Chronic low back pain	47.05	44/56	Therapeutic exercise	The physiotherapist supervises the patient in a therapeutic exercise programme consisting of movement control, stretching and aerobic exercises, 4–6 participants, 30–50 min 2 times/month, 3months.	ST	TSK-11
				Therapeutic exercise and pain neurophysiology education	The physiotherapist supervises the patient in a therapeutic exercise programme consisting of movement control, stretching and aerobic exercises+ pain neurophysiology education, 4–6 participants, 30–50 min, 2 times/month, 3 months.	MP	
Sarig Bahat et al. (2018)	Chronic neck pain	47.42	63/90	Training with VR devices	Training with VR devices, 5 min/time, 4 times a day, 4 times a week, 4 weeks.	MP	TSK-17
				Training with head-laser beam devices	Training with head-laser beam devices, 5 min/time, 4 times a day, 4 times a week, 4 weeks.	MP	
				No treatment	No treatment.	Blank	
Hott et al. (2019)	Patellofemoral Pain	27.60	73/113	Traditional knee-focused exercise	Patients performed supine straight leg raises, supine terminal knee extensions, and mini squats with back supported against the wall, 3 sets of 10 repetitions, 3/week for 6 weeks, under the supervision of a physiotherapist.	ST	TSK-13
				Hip-focused exercise	Patients performed lateral hip abduction, external hip rotation and prone hip abduction in 3 sets of 10 sessions, 3 sessions/week for 6 weeks under the supervision of a physiotherapist.	ST	
				Free physical activity	The physiotherapist supervises the patient's active choice of free movement for 6 weeks.	ST	
Gholami et al. (2020)	Rupture of the anterior cruciate ligament	32.30	2/20	Kinesio tape	5-cm Kinesio Tape length with 50% elongation.	ED	TSK-17
				Placebo KT group	Shame taping without tension in the tape.	Placebo	
Gulsen et al. (2020)	Fibromyalgia	41.38	16/16	Combined exercise	Patients initiate comprehensive exercise training, including 30 min of aerobic training and 30 min of Pilates training. 2/week, 8 weeks.	APE	TSK-17
				Combined exercise with VR devices	A combined exercise training with VR consisting of 30-min aerobic training and 30-min strengthening and flexibility exercises, 2/week, 8 weeks.	MP	
Hott et al. (2020)	Patellofemoral pain	27.60	73/112	Traditional knee-focused exercise	Straight-leg raises in the supine position, supine terminal knee extensions, and a mini-squat with the back supported against the wall, 3 sets, 10 repetitions, 3/week, 6 weeks were performed for 6 weeks.	APE	TSK-13
				Hip-focused exercise	Side-lying hip abduction, hip external rotation, and prone hip extension, 3 sets, 10 repetitions, 3/week, 6 weeks were performed for 6 weeks	APE	
				Free physical activity	Some physical education activities under the supervision of a physiotherapist.	ST	
Meirelles et al. (2020)	Chronic non-specific low back pain	48.00	28/38	Active Control	Therapeutic exercises under the supervision of a physiotherapist, 2/week, 10 weeks.	ST	TSK-17
				Active Control +Osteopathic Manipulation	Physiotherapist intervention with active control group and orthopedic manipulation techniques with OMTG, 30–45 min, 1/week, 5 weeks.	MP	
Zdziarski-Horodyski et al. (2020)	Orthopedic trauma	43.00	40/99	Integrative care	Routine care combined with a social self-support programme guided by orthopedic surgeons and care teams in 1 year.	MP	TSK-11
				Usual care	Regular medication under the supervision of a physiotherapist in 1 year.	PM	
Selhorst et al. (2021)	Patellofemoral pain	14.80	43/65	Psychologically Informed Intervention	The experimental group watched a short psychologically informed video (8 min and 30 s) targeting beliefs about pain related fear and pain catastrophizing.	PI	TSK-11
				Control video	Participants in the control group watched a video equal in length to the psychologically informed video (8 min and 30 s). The control video discussed basic anatomy of the lower extremity and the theorized biomedical factors involved in PFP.	TAU	
Gül et al. (2021)	Chronic low back pain	42.30	26/31	Physiotherapy combined with TNE	A physiotherapy programme of 15 sessions supervised by a physiotherapist and a home exercise programme for their 3 weeks.	MP	TSK-17
				Physiotherapy alone	A 15-session physiotherapy programme supervised by a physiotherapist for 3 weeks.	PM	

*TSK, Tampa scale of kinesiophobia; APE, Active Physical Exercise; ST, Supervised training; PI, Psychological Intervention; MP, Multi-modal Protocols; PM, Passive Modalities; ED, External-used Devices; TAU, Treatment as Usual*.

### Risk of Bias

**Figure 2:**
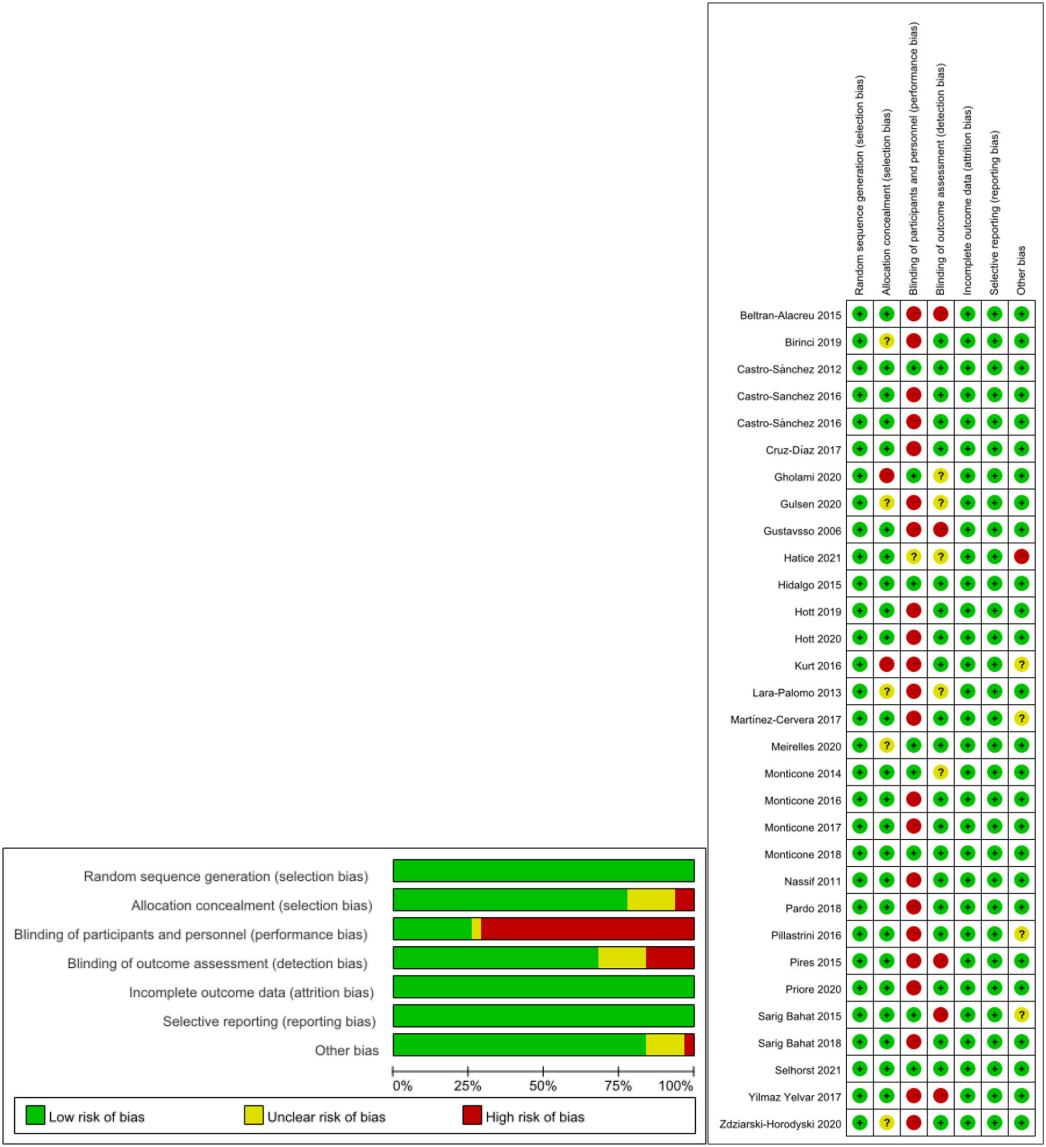
The result of the risk of bias assessment. **(A)** Risk of bias summary; **(B)** Risk of bias graph.

The risk of bias for the 31 included studies was assessed in this review and the results are shown in Figure 2. Adequate results were obtained for the randomization and concealment methods of the participants mentioned in the studies. Eight studies adequately described participants and staff as blind, and a total of 21 studies described assessors as blind. The risk of attrition bias was low (low in all studies), the risk of selection bias was low (low in all studies), and the risk of reporting bias was low (low in all studies).

### Network Meta-Analysis

#### Network Geometries

**Figure 3:**
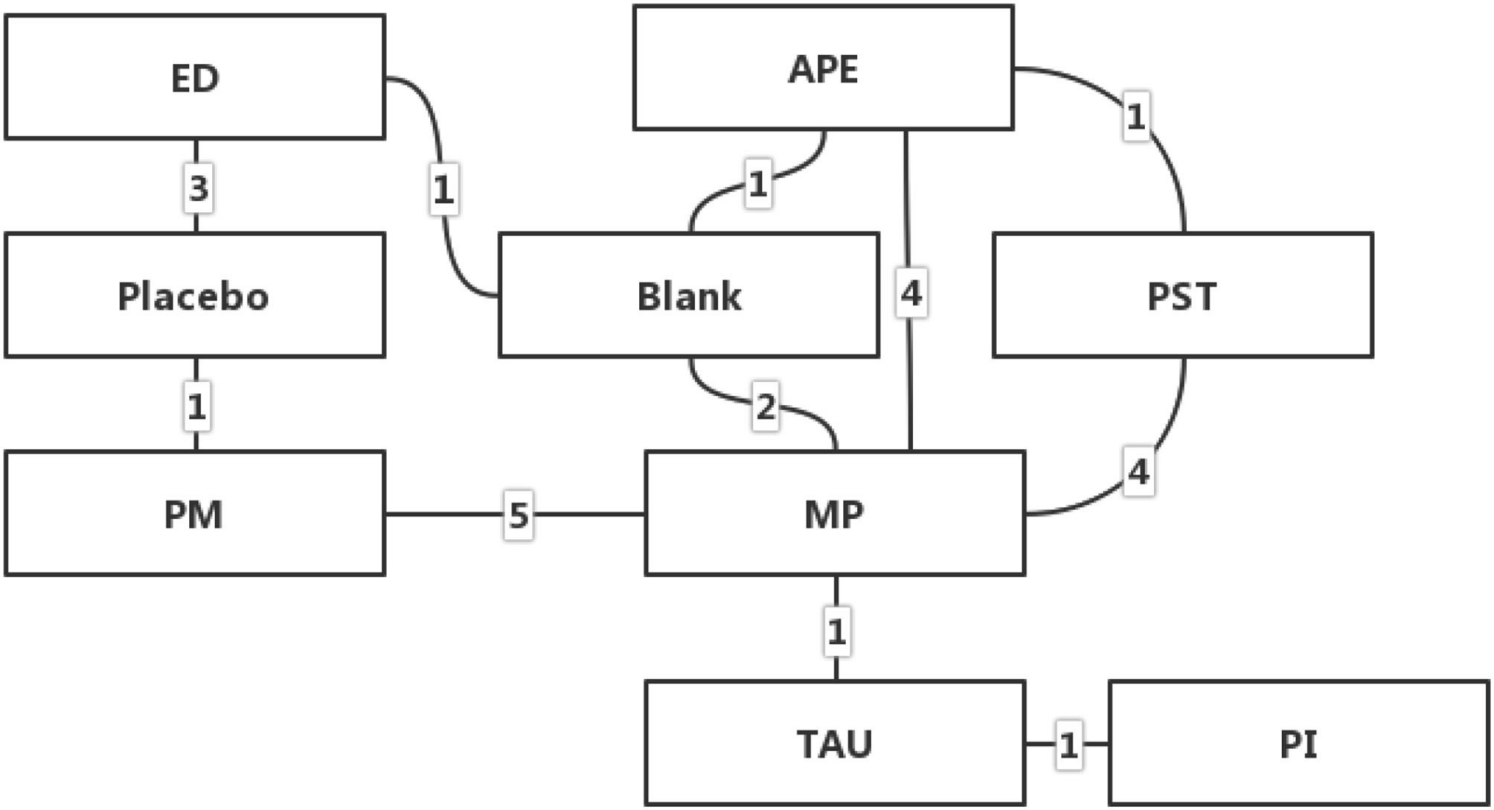
Network geometry of the interventions (APE, Active Physical Exercise; ST, Supervised Training; PI, Psychological Intervention; MP, Multi-modal Protocols; PM, Passive Modalities; ED, External-used Devices; TAU, Treatment as Usual; These figures represent the number of studies that have made direct comparisons between interventions).

The network geometries showed all categories of treatment protocols, providing information such as the type of treatment represented by each node, the direct comparisons between each pair of interventions represented by the lines, and the arms of each trial represented by the number on the edges. The network geometries of the interventions are presented in Figure 3.

#### Comparisons of Random Effects Standard Deviations

The results of the random effects standard deviation calculations in both the consistency model and inconsistency model of each outcome measure are provided in Table 2. According to the results, the random effects standard deviations of the consistency modal were identical. Therefore, the analysis under the consistency model had a good validity.

**Table 2:** Random effect standard deviation and 95% confidence interval value of the agreement model and the inconsistency model.

**Random effects standard deviation**	**Median (95% CI)**
Consistency model	4.94 (3.38, 7.78)
Inconsistency model	4.54 (2.83, 7.48)

#### Results of Network Meta-Analysis

**Figure 4:**
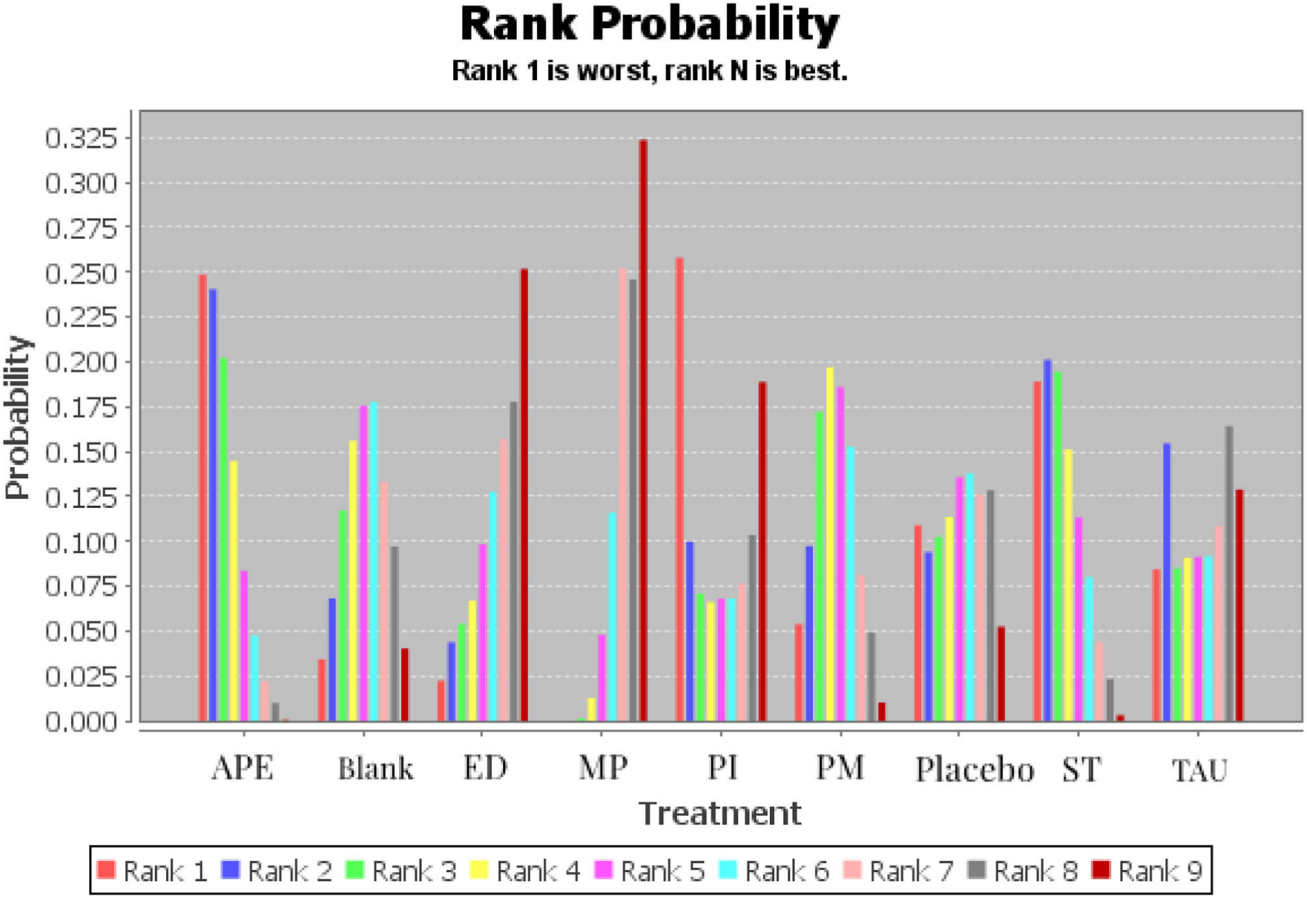
Ranking of measures and probabilities (APE, Active Physical Exercise; ST, Supervised training; PI, Psychological Intervention; MP, Multi-modal Protocols; PM, Passive Modalities; ED, External-used Devices; TAU, Treatment as Usual).

Table 3 shows the league tables of the network geometries, and the ranking of measures and probabilities is provided in Table 4 and Figure 4. Since a lower score of TSK is regarded as better, the treatment protocol in Rank 1 has the highest probability to be the last treatment choice, whereas that in Rank 9 has the lowest probability to be the first treatment choice.

**Table 3:** League tables of the network geometries.

**APE**								
3.31 (−3.36, 10.16)	**Blank**							
5.62 (−4.51, 15.49)	2.26 (−6.44, 11.23)	**ED**						
6.76 (1.90, 11.38)*	3.49 (−2.81, 9.49)	1.20 (−8.15, 10.45)	**MP**					
2.62(−14.01, 19.26)	−0.75(−17.84, 16.61)	−2.93 (−21.86, 15.20)	−4.13(−20.10, 12.03)	**PI**				
2.34 (−4.54, 9.03)	−0.92 (−8.63, 6.41)	−3.20 (−12.66, 5.91)	−4.47 (−9.26, 0.43)	−0.26 (−17.26, 16.30)	**PM**			
3.31 (−7.22, 13.37)	−0.05 (−9.43, 9.08)	−2.33 (−8.27, 3.45)	−3.53(−12.89, 5.75)	0.63 (−17.98, 19.11)	0.83 (−7.99, 10.04)	**Placebo**		
0.62(−5.68, 6.80)	−2.63 (−10.50, 4.91)	−4.85 (−15.58, 5.40)	−6.10 (−11.27, −1.10)*	−1.93 (−18.84, 14.39)	−1.74 (−8.90, 5.42)	−2.58 (−13.38, 7.87)	**ST**	
3.56(−9.03, 16.29)	0.30 (−13.13, 13.48)	−1.99 (−16.93, 13.02)	−3.22 (−14.97, 8.70)	0.93 (−9.91, 11.65)	1.20 (−11.44, 14.15)	0.32(−14.77, 15.47)	2.87(−9.95, 15.83)	**TAU**

*APE, Active Physical Exercise; ED, External-used Devices; MP, Multi-modal Protocols; PI, Psychological Intervention; PM, Passive Modalities; ST, Supervised training; TAU, Treatment as Usual.*

*p <0.05.

**Table 4:** Ranking of measures and probabilities.

**Treatment**	**Rank 1**	**Rank 2**	**Rank 3**	**Rank 4**	**Rank 5**	**Rank 6**	**Rank 7**	**Rank 8**	**Rank 9**
Active physical exercise	0.25	0.24	0.20	0.14	0.08	0.05	0.02	0.01	0.00
Blank	0.03	0.07	0.12	0.16	0.18	0.18	0.13	0.10	0.04
Multi-modal protocols	0.00	0.00	0.00	0.01	0.05	0.12	0.25	0.25	0.32
Passive modalities	0.05	0.10	0.17	0.20	0.19	0.15	0.08	0.05	0.01
Supervised training	0.19	0.20	0.19	0.15	0.11	0.08	0.04	0.02	0.00
External-used devices	0.02	0.04	0.05	0.07	0.10	0.13	0.16	0.18	0.25
Psychological intervention	0.26	0.10	0.07	0.07	0.07	0.07	0.08	0.10	0.19
Treatment as usual	0.08	0.15	0.09	0.09	0.09	0.09	0.11	0.16	0.13
Placebo	0.11	0.09	0.10	0.11	0.14	0.14	0.13	0.13	0.05

According to the result, MP had the highest potential probability to be the best choice in treating kinesiophobia caused by musculoskeletal pain (0.32 in Rank 9 and 0.25 in Rank 8) with a statistically significant advantage in the comparison with APE (*P* < 0.05), whereas ED might have the highest potential probability to be the second-best treatment choice with a probability of 0.25 in Rank 9 and 0.18 in Rank 8. The psychological intervention probability is the third-best treatment choice with a probability of 0.19 in Rank 9 and 0.10 in Rank 8. Corresponding with the results of the included studies, TAU and Blank, which were usually set as interventions in the control group, have a high potential probability in Ranks 1 and 2. Nevertheless, APE has the highest potential probability in Ranks 1 and 2.

## Discussion

The objective of this systematic review and network meta-analysis is to make a mixed comparison of intervention treatment protocols for kinesiophobia caused by musculoskeletal pain. After comparing 9 different categories of treatment protocols, the primary findings are as follows. First, multi-modal protocols have the highest potential probability to be the best choice in treating kinesiophobia caused by musculoskeletal pain and have a statistically significant advantage in the comparison with active physical exercise and supervised training. Second, wearing an external-used device have the highest potential probability to be the second-best treatment choice. Third, keeping active in physical exercise might not be a wise choice when the patient has been diagnosed with kinesiophobia induced by musculoskeletal pain.

Multi-model treatment protocols seem to have the highest potential probability to be the best treatment choice. This result could be supported by high-level clinical evidence. Apart from the systematic and meta-analysis mentioned in the introduction section of this review (Xu et al., 2020), many randomized controlled trials concluded a similar result to support the clinical practice of multi-disciplinary interventions for kinesiophobia caused by musculoskeletal pain. For example, a pragmatic randomized controlled trial conducted in 2020 claimed that a multi-model treatment protocol combined with nature activity therapy and usual care could be an effective co-adjuvant multicomponent treatment for improving fibromyalgia-related symptoms (Serrat et al., 2020), and another randomized controlled trial conducted in 2013 demonstrated that a long-term multidisciplinary treatment program was superior to the exercise program in reducing disability, fear-avoidance beliefs and pain, as well as enhancing the quality of life of patients with chronic low back pain. The effects were clinically tangible and lasted for at least 1 year after the program ended (Monticone et al., 2013). Moreover, from the non-clinical perspective, a review conducted in 2006, which determined the effectiveness and cost-effectiveness of return-to-work outpatient multidisciplinary treatment programs for sick-listed workers with non-specific upper extremity musculoskeletal complaints, found that multidisciplinary treatment affects individuals positively, but shows no significant difference in cost-effectiveness on the societal level as compared to usual care (Meijer et al., 2006).

The multi-modal treatment protocols included in the review referred to approaches from two or more of the following perspectives, which were physiological, and psychological perspectives. The results of the network meta-analysis showed that the effect of multi-modal treatment protocols was better than that of treatment protocols from only a physiological perspective. This result is correspondent with some previous studies. For example, in a review published in 2014, the therapist performed spinal stabilization exercises based on cognitive behavioral therapy for the patient, and the results showed that spinal stabilization exercises based on cognitive behavioral therapy could bring more advantages to the patient when they were performing non-spinal motor tasks such as walking (Monticone et al., 2014). Another review conducted in 2018 demonstrated that patients with chronic low back pain who were given a pain education session by a psychologist after supervised training by a physiotherapist could feel less pain than those who only had supervised training (Pardo et al., 2018). The results of the network meta-analysis also showed that all multi-modal treatment protocols of the included studies contained treatments from a psychological perspective, indicating that psychological intervention has a potentially positive effect on the kinesiophobia caused by musculoskeletal pain. There is one review included in this study that compares psychological interventions with TAU and finds a significant advantage of psychological intervention over TAU in reducing the severity of kinesiophobia (Selhorst et al., 2021). The results of some previous studies identified the advantage of psychological treatment protocols in treating kinesiophobia induced by musculoskeletal pain and demonstrated that psychological treatment could relieve kinesiophobia caused by physical disorders and improve quality of life (Goudie et al., 2018; Innes et al., 2018; Helminen et al., 2020; Serrat et al., 2020; Xu et al., 2022). For example, a review published in 2016, which has also been included in this systematic review, demonstrated that group-based and task-oriented exercises could reduce disability, kinesiophobia, catastrophizing, and enhance the quality of life of individuals with chronic low back pain (Monticone et al., 2016). Moreover, a review by Wicksell's team, which is a secondary analysis of a randomized controlled trial, resulted in that cognitive behavior therapy could improve the functioning and life satisfaction of people with chronic pain (Wicksell et al., 2010). The potential mechanism of psychological treatments might come from self-efficacy, a review conducted in 2020 shows both pain self-efficacy and negatively charged emotion and expectations toward pain are important factors when dealing with musculoskeletal injured patients (Sinikallio et al., 2014). However, some previous studies concluded different views, a review conducted in 2006 compared the effectiveness of a brief physiotherapy pain management approach using cognitive-behavioral principles with a commonly used method of passive modalities, and identified that the latter approach resulted in higher patient satisfaction overall but the former could be more cost-effective (Moffett et al., 2006). The heterogeneity of the results might be caused by the different expectations of patients when they are selecting treatment protocols. Future studies should compare the effects of different psychological intervention protocols in a particular population to identify the effectiveness of kinesiophobia.

Besides, wearing an externally used device has a high potential probability to be the second-best treatment choice. There are 4 relevant studies included in this review, and 3 studies are randomized controlled trials about the effect of Kinesio tape. For example, a review in 2012 explored the effect of Kinesio tape adhered in the lumbar area, finding that a 1-week treatment of Kinesio tape had a positive effect on relieving pain than the placebo tape (Castro-Sánchez et al., 2012). A review conducted in 2016 evaluated the short-term effects of Kinesio tape on joint position sense, isokinetic measurements, kinesiophobia, symptoms, and functional limitations in patients with patellofemoral pain syndrome, demonstrating that, although short-term Kinesio tape application could not increase hamstring muscle strength, it still could improve joint position sense, pain, kinesiophobia, symptoms, and daily limitations (Kurt et al., 2016). The positive effect of the Kinesio tape application has been identified by several previous studies (Coombes et al., 2016; Karran et al., 2017; Louw et al., 2017; Takacs et al., 2017); however, most of these studies only identified the short-term effect of Kinesio tape. Further studies should identify the long-term effect of Kinesio tape and that of other kinds of externally used therapy devices.

What should also be noticed among the results of the network meta-analysis is that, for the treatment of kinesiophobia caused by musculoskeletal pain, active physical exercise might not be recommended with the lowest potential, which seems even lower than placebo, within the 9 treatment protocol categories. The reason might come from two possible aspects. First, active physical exercise might lead to secondary injury and further pain. Second, this result might be caused by the heterogeneity of intervention protocols in these included studies. Therefore, the interpretation of this result should be cautious, and future studies should explore the effect of active physical exercise on kinesiophobia without covariate interference.

Within all 9 categories of treatment protocols, TAU and Blank, which were usually set as interventions in the control group, have a low potential probability to have a positive effect on kinesiophobia caused by musculoskeletal pain. It is because treatment as usual and no-treatment, themselves are not clinical treatment protocols, only being set as interventions in control groups in the included studies. Besides, this result could also indicate that kinesiophobia might not be a self-limiting disease whose symptoms would not resolve themselves over time. It means that if patients with kinesiophobia do not carry out a particular treatment, only usual care would not be effective on their symptoms.

The primary limitation of this systematic review is that there is a limited number of studies that focus on the treatments for kinesiophobia, leading to a lack of arms in some direct intervention comparisons. For example, there is no direct comparison between psychological intervention and externally used devices in the network geometry. Last but not least, the score of TSK is usually reported as a secondary outcome, resulting in publication bias to some extent (Bennell et al., 2020; Öztürk et al., 2021; Serrat et al., 2021).

## Conclusion

Multi-modal protocols could be recommended as the preferred option when dealing with kinesiophobia caused by musculoskeletal pain. However, it is still worth mentioning that there are also potentially positive therapeutic effects of psychological interventions. Since the concept of kinesiophobia is based on the fear-avoidance model, the psychological mechanism should be paid enough attention to during treatment.

## Data Availability Statement

The original contributions presented in the study are included in the article/Supplementary Materials, further inquiries can be directed to the corresponding authors.

## Author Contributions

JH conceived the study design, collected and analyzed data, and drafted the manuscript. YX and RX participated in the design of the study, collected, and analyzed data. YG, RX, and JB conceived the study design, assisted in revising the manuscript, and reviewed the first and final versions of the manuscript. All authors contributed to the article and agreed to the submitted version of the manuscript.

## Funding

This study was supported by Zhejiang Province Medical and Health Science and Technology Plan Project (No. 2018KY710), Ningbo Public Welfare Science and Technology Plan Project (No. 2019C50095), Health Youth Technical Talent Cultivation Special Fund Project (2020SWSQNGG-01), Ningbo Medical Science and Technology Plan (2020Y14), and Young Cultivation Fund Project of the Affiliated of School of Medicine of Ningbo University (FYQM-KY-202003).

## Conflict of Interest

The authors declare that the research was conducted in the absence of any commercial or financial relationships that could be construed as a potential conflict of interest.

## Publisher's Note

All claims expressed in this article are solely those of the authors and do not necessarily represent those of their affiliated organizations, or those of the publisher, the editors and the reviewers. Any product that may be evaluated in this article, or claim that may be made by its manufacturer, is not guaranteed or endorsed by the publisher.
